# Integrative multi-omics analysis reveals probiotic-induced microbiota shifts in women with gestational diabetes

**DOI:** 10.3389/fcimb.2026.1782744

**Published:** 2026-03-16

**Authors:** Xiaohua Su, Jindi Yang, Zhen Le, Jingwen Xiao, Daoyan Zhao

**Affiliations:** 1 Department of Obstetrics, Guangdong Women and Children Hospital, Guangzhou, Guangdong, China; 2 Department of Gynecology, The Fifth Affiliated Hospital of Southern Medical University, Guangzhou, Guangdong, China; 3 Department of Obstetrics, The Fifth Affiliated Hospital of Southern Medical University, Guangzhou, Guangdong, China

**Keywords:** gestational diabetes mellitus (GDM), gut barrier function, gut microbiota, microbial metabolite-host interactions, multi-omics integration, probiotics

## Abstract

**Introduction:**

Gestational diabetes mellitus (GDM) is a common pregnancy disorder. It is associated with impaired glucose tolerance and insulin resistance, increasing the potential risks for both maternal and fetal complications. GDM is associated with an increased risk of type 2 diabetes later in life. Management is a big issue in maternal health. New work has underscored the role of the gut microbiota in metabolism and immune function. This indicates that probiotics might exert their mode of action through modulating the microbiota and controlling metabolism.

**Methods:**

This study employs a multi-omics strategy to assess the impact of probiotic administration on gut microbiota composition, metabolomic profiles, and host gene expression in GDM women. Women with GDM received probiotics for 8 weeks. Metagenomic sequencing quantified alterations of gut microbiota composition and LC-MS provided untargeted metabolomics in serum and urine. Gene expression was analyzed by qRT-PCR in reference to other physiological factors such as insulin signaling, inflammation, oxidative stress, and gut barrier. Data integration was performed using Principal Component Analysis (PCA), Partial Least Squares Discriminant Analysis (PLS-DA), and network analysis, then pathway enrichment analysis was conducted with KEGG and MetaboAnalyst.

**Results:**

The supplementation of probiotics resulted in a significant change of gut microbiota (Lactobacillus 7.6-fold; Bifidobacterium 6.4-fold). Escherichia/Shigella was reduced. The amounts of short-chain fatty acids (SCFAs), especially butyrate and acetate, were increased 3.1 fold and 2.5 fold, respectively. In a gene expression assessment, the insulin receptor and AKT increased 2.5- and 1.9-fold higher, respectively, indicating greater insulin sensitivity. Levels of TNF-α and IL-6 decreased; however, genes related to gut barrier function (ZO-1, CLDN1) increased.

**Discussion:**

The administration of probiotic has a great impact on gut microbiome, metabolic activity, and host gene expression in women with GDM. Our data indicate that probiotics may represent a non-invasive and safe treatment for gestational diabetes through enhancing insulin sensitivity, anti-inflammatory environment, and gut health status. Larger confirmatory studies are needed to corroborate these findings and augment future clinical application of probiotics in GDM patients.

## 1 Introduction

Gestational Diabetes Mellitus (GDM) is a gestation-associated disease with impaired glucose tolerance (Modzelewski et al., 2022). It impacts 2–10% of pregnancies worldwide, making it one of the most frequent issues among pregnant women (Natarajan et al., 2023). GDM is related to greater risk of maternal type 2 diabetes, hypertension, and preeclampsia. It has also been linked to adverse infant outcomes such as macrosomia, preterm birth, and neonatal hypoglycemia. While GDM typically goes away after a woman gives birth, those who have had it are at increased risk for developing type 2 diabetes. They are also more likely to have metabolic disturbances when they grow up (Malaza et al., 2022).

Gestational diabetes is a multifactorial disorder in which insulin resistance, low-grade inflammatory state, oxidative stress and alterations in gut microbiota contribute to its pathogenesis (Saucedo et al., 2023). Respectively, insulin resistance increases during pregnancy naturally by human placental lactogen and cortisol, which may induce glucose intolerance (Leoni et al., 2022). However, compensatory increase in insulin production is inadequate in women with GDM causing hyperglycemia (Usman et al., 2023). Another critical characteristic of GDM is chronic low-grade inflammation, which leads to insulin resistance and endothelial dysfunction and affects glucose and lipids metabolism (Xuan Nguyen et al., 2023).

In addition to these pathways, emerging evidence indicates that the gut microbiota, or bacteria colonizing in the gastrointestinal tract, has an influence on GDM development and progression (Di Vincenzo et al., 2024). The gut microbiome is involved in several physiological and functional activities, such as digestion, immunological regulation, metabolism and intestinal barrier functions. Dysbiosis is connected to various metabolic diseases, such as obesity and insulin resistance, associated with type 2 diabetes (Patra et al., 2023). A few past investigations have reported alterations in the gut microbiota composition during gestational diabetes characterized by a decrease in diversity and increase of pro-inflammatory microorganisms leading to further insulin resistance and systemic inflammation (Yan et al., 2023).

As the effect of microbiome on metabolic health has now been better appreciated, there is an increasing interest in applying probiotics as a novel intervention for GDM (Kamińska et al., 2022). Probiotics refers to live bacteria with a positive impact on your health if you ingest enough of them especially for gut health and immune regulation. Probiotics assist in regulating the microbiota of the gut, decreasing systemic inflammation and ameliorating symptoms related to metabolism as occur from IBS, obesity and type 2 diabetes. The advantages of gestational diabetes are, however, still obscure, and the way by which such would work in pregnancy is also unknown (Mazziotta et al., 2023).

In gestational diabetes, probiotics might be particularly useful. They can alter gut microbiota, increase insulin sensitivity and regulate inflammation (Yefet et al., 2023). These Probiotics boost the number of good bacteria in your system, including Lactobacillus and Bifidobatterium. These are associated with anti-inflammatory properties and gut barrier enhancement (de Albuquerque Lemos et al., 2024). These bacteria beget a number of metabolites including butyrate, acetate, and propionate. These metabolites are beneficial for improving insulin sensitivity, and alleviating inflammation as well as intestinal barrier (Zhang et al., 2023).

The anti-inflammatory effect of probiotics in gestational diabetes is also becoming more popular. The GDM is associated with chronic inflammation, which involves functional and quantitative changes in pro-inflammatory cytokines such as TNF-α, IL-6 levels that promote insulin resistance and endothelial dysfunction (de Mendonça et al., 2022). Probiotics may also reduce systemic inflammation by increasing anti-inflammatory cytokine levels (IL-10 and TGF-β). Moreover, the role of oxidative stress in GDM development is well known. Reactive oxygen species (ROS) participate in insulin resistance and beta cell dysfunction in the pancreas. It has been demonstrated that probiotics can enhance antioxidant defense systems, leading to a decrease in oxidative stress and an improvement in glucose metabolism (Viana et al., 2025).

State-of-the-art mult-omics technologies using metagenomic, metabolomic and transcriptomic data have enabled to the ability to dissect complex bacterial-metabolite-host relationships (Satya et al., 2024). Metagenomics reveals the composition and diversity of gut microbial communities, while metabolomics unveils metabolic outputs that result from these microbial communities., 2023), the former allowing for the study of host gene expression and how probiotics could influence gene regulation related to insulin sensitivity, inflammation and oxidative stress (Lee et al., 2023).

Multi-omics inclusion has offered a more complete overview of microbe-metabolite-host interaction, which further facilitates the comprehension on how probiotics rescue the disturbed metabolic processes and the disrupted insulin signaling, immunological balance in gestational diabetes (Kwoji et al., 2023). This integrative perspective allows the identification of central regulatory hubs in both gut microbiota, metabolic pathways, and host genes responsive to probiotic supplementation, resulting in a model description that comprehensively captures the potential impact of probiotics on women suffering from gestational diabetes.

Although several clinical and experimental studies have explored probiotic supplementation in gestational diabetes mellitus, most investigations have primarily focused on glycemic indices, inflammatory biomarkers, or microbiota compositional shifts in isolation. Limited efforts have been made to integrate microbial, metabolic, and host transcriptomic responses to elucidate the systemic mechanisms underlying probiotic efficacy in GDM. Furthermore, existing reports often rely on single-omics or targeted analyses, thereby restricting the comprehensive understanding of microbe–metabolite–host signaling networks. To address these limitations, the present study adopts an integrative multi-omics framework combining metagenomic sequencing, untargeted metabolomics, and host gene expression profiling. This systems-level strategy enables the identification of coordinated microbial and host regulatory pathways modulated by probiotic intervention, thereby offering mechanistic insights beyond conventional probiotic efficacy studies.

The probiotic strains selected for this study were primarily members of the *Lactobacillus* and *Bifidobacterium* genera, chosen based on their well-documented roles in metabolic and immunological regulation. These taxa are among the most extensively studied probiotics in metabolic disorders, including GDM, due to their ability to enhance short-chain fatty acid (SCFA) production, particularly butyrate and acetate, which are known to improve insulin sensitivity and glucose metabolism. In addition, *Lactobacillus* species contribute to gut barrier stabilization through tight-junction protein modulation, while *Bifidobacterium* species exert anti-inflammatory effects by suppressing pro-inflammatory cytokines such as TNF-α and IL-6. Both genera are also involved in bile acid transformation, oxidative stress reduction, and modulation of host insulin signaling pathways. Therefore, their selection was mechanistically aligned with the metabolic, inflammatory, and gut permeability abnormalities characteristic of gestational diabetes mellitus.

The goal of this study is to:

1. Apply metagenomic sequencing to investigate the impact of probiotic intervention on gut microbiota in women with GDM.
2. Apply liquid chromatography-mass spectrometry (LC-MS) to identify metabolite profiles and pathway metabolism after administration of probiotics.
3. Assess insulin signaling, inflammation, oxidative stress and gut barrier function of host genes using qRT-PCR.
4. Using multi-omics analysis to reveal the effect of probiotics on microbe-metabolite-host interactions and pathways to better understand their applicability for gestational diabetes.

In general, the current study aims to elucidate molecular basis of probiotic benefits on gestational diabetes and potential specific microbial strains, metabolic pathways and host responses which could enhance gestational diabetes treatment with a long-term perspective on preventing risks for mothers and offspring.

## 2 Materials and methods

### 2.1 Sample preparation for multi-omics profiling

Participants received an oral multi-strain probiotic capsule containing *Lactobacillus acidophilus*, *Lactobacillus rhamnosus*, *Bifidobacterium bifidum*, and *Bifidobacterium longum*, with a total viable count of 1 × 10¹^0^ CFU per capsule. Subjects were instructed to consume one capsule twice daily for a period of 12 weeks. Compliance and tolerance were monitored through scheduled follow-ups. These strains were obtained as part of a commercially available probiotic preparation approved for human consumption and widely used in clinical and nutritional supplementation. Therefore, the strains used in this study were not laboratory isolates but standardized commercial strains with validated safety and viability profiles. Fecal, blood and urine samples collected at baseline and after treatment were independently analyzed for metagenomics, metabolome and transcriptome. We used fecal samples for microbial DNA extraction, and blood samples were processed to obtain host RNA and serum metabolites (and urine samples for additional profiling of the metabolome). For stabilization of the molecular integrity, samples were all kept at −80 °C until analysis was performed.

### 2.2 Metagenomic analysis of gut microbiota

#### 2.2.1 DNA extraction and sequencing

Microbiota DNA was extracted from fecal samples with a stool DNA extraction kit (Qiagen) according to the manufacturer’s protocol. The V3–V4 region of the bacterial 16S rRNA gene was amplified using universal primers and pair-end sequenced using Illumina MiSeq.

#### 2.2.2 Bioinformatics processing

Sequencing reads were first pre-processed as follows using the QIIME2 pipeline. Quality filtering, denoising, chimera removal, and amplicon sequence variant calling were conducted using the DADA2 algorithm. The classification was done taxonomically with SILVA reference database.

#### 2.2.3 Microbial diversity and functional prediction

We calculated the abundance-based alpha (Shannon, Simpson and Chao1) diversity and beta diversity (Bray-Curtis dissimilarity) index for the microbial community as described previously. A differential abundance analysis was used to determine taxa correlated with probiotics. The potential function of the microbial community was predicted based on PICRUSt2 for metabolic pathways and gene functions.

### 2.3 Metabolomic analysis

#### 2.3.1 Metabolite extraction

Precipitation with methanol (protein precipitation) at 4 °C was performed to generate serum and urine samples, which were subsequently centrifuged and filtered. The metabolite-rich supernatant was collected and analyzed.

#### 2.3.2 LC-MS data acquisition

LC-MS untargeted metabolomic analysis Liquid chromatography-mass spectrometry (LC-MS) was conducted. Quantified and identified metabolites Separation of metabolites was achieved on a reverse-phase C18 column using both positive and negative ion modes. Raw LC-MS data, such as peak detection, alignment, normalization and scaling, were analyzed by MetaboAnalyst. Metabolites were also identified using the Human Metabolome Database (HMDB), METLIN, and KEGG databases. Metabolic effects of probiotic treatment were further analyzed by differential metabolite analysis.

### 2.4 Host transcriptomic analysis

#### 2.4.1 RNA extraction and cDNA synthesis

Peripheral blood mononuclear cells (PBMCs) were separated from whole-blood samples. RNA extraction, quantification and cDNA synthesis Total RNA was extracted, quantified and reverse transcribed into cDNA.

#### 2.4.2 Gene expression analysis

The expression of genes related to insulin signaling, inflammation, immunomodulation, oxidative stress and gut barrier function were evaluated by qRT-PCR. Quantitative real-time PCR (qRT-PCR) amplification was performed using a standard SYBR Green chemistry protocol on a real-time PCR detection system. The thermal cycling conditions were as follows: initial denaturation at 95 °C for 5 min to activate DNA polymerase, followed by 40 amplification cycles consisting of denaturation at 95 °C for 15 s, primer annealing at 58–60 °C for 30 s (gene-specific optimization), and extension at 72 °C for 30 s. Fluorescence signals were recorded at the end of each amplification cycle. A melt-curve analysis was conducted from 65 °C to 95 °C to verify amplification specificity and exclude primer-dimer formation. All reactions were performed in triplicate, and relative gene expression levels were calculated using the 2^-^ΔΔCt method with β-actin as the internal housekeeping control. The forward and reverse primer sequences used for qRT-PCR amplification of all target genes are listed in **Supplementary Table 1**.

### 2.5 *In silico* multi-omics data integration

#### 2.5.1 Data normalization and scaling

Metagenomic, metabolomic and transcriptome data were level-shifted and variance scaled to enforce layer consistency across omics datasets.

#### 2.5.2 Multivariate and network analysis

Global features and clustering of multi-omics data were analyzed by principal component analysis (PCA) and partial least squares discriminant analysis  (PLS-DA). To enable integrative dimensionality reduction and cross-omics correlation exploration, multivariate statistical modeling was performed using the mixOmics package implemented in R (v4.2.0). Sparse Partial Least Squares Discriminant Analysis (sPLS-DA) was applied to identify discriminative features across omics layers contributing to probiotic intervention effects. Model robustness was assessed using 10-fold cross-validation and permutation testing (n = 1,000 permutations). Variable Importance in Projection (VIP) scores were calculated to prioritize key microbial taxa, metabolites, and genes driving group separation. Hierarchical clustering with Euclidean distance and Ward linkage was additionally performed to visualize co-regulated feature clusters across datasets.

Microbe-metabolite-host interaction networks were generated through correlation-based network analysis and identified key regulatory hubs regulated by the probiotic intervention. Correlation-based integrative network analysis was conducted to elucidate functional relationships between microbial taxa, metabolites, and host genes. Pairwise correlations were computed using Spearman’s rank correlation coefficient to accommodate non-parametric data distributions. Associations with correlation coefficients |r| ≥ 0.6 and false discovery rate (FDR)-adjusted p-values < 0.05 were considered statistically significant. The resulting association matrices were imported into Cytoscape (v3.9.1) for network visualization and topological analysis. NetworkAnalyzer was used to compute node centrality parameters including degree, betweenness centrality, and clustering coefficient to identify key regulatory hubs. Microbial taxa exhibiting high connectivity with metabolic and transcriptomic nodes were designated as keystone probiotic-responsive regulators. Positive correlations were interpreted as cooperative functional relationships (e.g., SCFA-producing microbes linked with anti-inflammatory gene expression), whereas negative correlations indicated antagonistic interactions, particularly between pathogenic taxa and beneficial metabolites.

### 2.6 Statistical analysis

The sample size was determined using G*Power statistical software based on an expected effect size of 0.8, with a significance level (α) of 0.05 and statistical power of 80%. The calculated minimum sample requirement was satisfied by the enrolled study cohort, ensuring adequate power to detect significant multi-omics differences following probiotic intervention. To mitigate inter-individual variability and pregnancy-related confounding, a paired longitudinal design (baseline vs. post-intervention) was employed, wherein each participant served as her own control.

All statistical analyses were conducted using R (v4.2.0), SPSS (v26.0), and MetaboAnalyst 5.0. Multi-omics visualization outputs including PCA plots, clustered heatmaps, and network diagrams were generated using ggplot2, mixOmics, and Cytoscape platforms. Multiple testing corrections were applied using the Benjamini–Hochberg false discovery rate method to ensure statistical rigor in integrative analyses.

## 3 Results

### 3.1 Metagenomic analysis of gut microbiota

#### 3.1.1 Sequencing quality and ASV generation

All samples generated a total of 5,000,000 paired-end reads, with an average of 250,000 reads per sample after quality filtering (Paired-end reads are sequences read from both ends of a DNA fragment, which helps with accuracy). Following denoising (sequencing errors removal) and chimera elimination (artificially formed hybrid sequences removed), 95% of the raw sequences were retained. In all 250 unique ASVs, that are the genotypes sequenced in the samples were successfully recovered. Rarefaction analysis (which determines if there was enough sequencing to capture the microbial diversity) confirmed a high depth of sequencing, as curves reached plateaus at 250,000 reads and this level appeared to include all the diversity in tested samples.

#### 3.1.2 Taxonomic composition of gut microbiota

At baseline, the dominant phyla were Firmicutes (38.5% ± 2.1%), Bacteroidota (30.7% ± 3.4%) and Actinobacteriota (9.2% ± 1.5%). The changes observed after the intervention were as follows: Actinobacteria increased to 12.8% ± 1.9% (p < 0.05); Firmicutes decreased to 35.6% ± 2.5% (p < 0.01) and; Proteobacteria declined from 6.5% ± 1% at baseline to 3.8% ± 1%.

**Figure 1:**
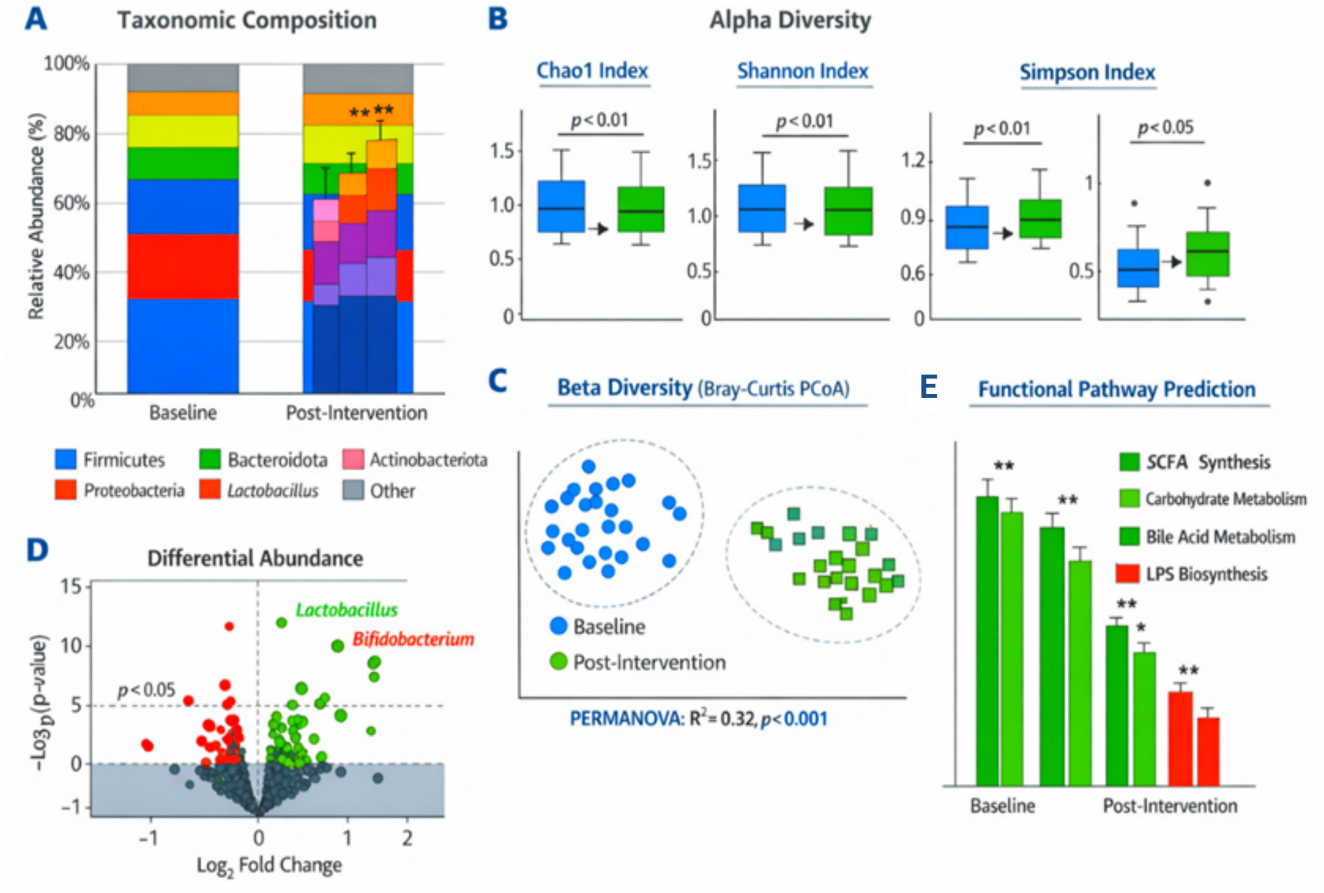
Metagenomic Analysis. **(A)** Taxonomic composition of the gut microbiota at baseline and post-intervention at the phylum and genus levels. **(B)** Alpha diversity indices (Shannon, Chao1, Simpson) for baseline and post-intervention samples. **(C)** Principal coordinate analysis (PCoA) based on Bray–Curtis dissimilarity to show separation between baseline and post-intervention samples. **(D)** Differential abundance of key microbial taxa **(E)** Predicted functional pathways enriched post-intervention. *Indicates that the result is statistically significant at p < 0.05. **Indicates that the result is highly statistically significant at p < 0.01.

Low proportions of beneficial bacteria, including Lactobacillus (0.8% ± 0.5%) and Bifidobacterium (0.5%± 0.4%) were registered for the baseline samples - which was extracted from the participants at Day 1 during Admission/On-Board sampling rounds. Addition of supplementation increased Lactobacillus to 6.1% ± 2.4% (p < 0.01) and Bifidobacterium to 3.2% ± 1.3%. In contrast, inflammation- and dysbiosis-associated taxa (here types of bacteria), such as Escherichjson/Shigella, were decreased from 4.9% ± 1.3% to 2.3% ± 1.0% (p <0.05). **Figure 1A** illustrates the relative abundance of dominant microbial taxa at baseline and post-intervention. Firmicutes and Bacteroidota were predominant at baseline, while probiotic supplementation increased Actinobacteriota and beneficial genera including *Lactobacillus* and *Bifidobacterium*, with a concurrent reduction in *Escherichia/Shigella*.

#### 3.1.3 Alpha diversity analysis

The intervention led to an evident increase in microbial richness. The Chao1 index value increased from 121.4 ± 18.3 at baseline to 157.2 ± 21.7 (p < 0.01), suggesting enhanced microbial diversity. The Shannon index was found to be higher than 3.9 ± 0.5 and increased significantly from 4.5 ± 0.6 (p < 0.01), suggesting a more even community and more species variety, for example). Simpson index (a dominance index) declined from 0.87 ± 0.02 at t = 0 to 0.79 ± 0.03 (p < 0.05), indicating a less dominant community structure post-supplementation (**Figure 1B**).

#### 3.1.4 Beta diversity analysis

Bray-Curtis-based beta-diversity analysis demonstrated that microbiome composition between baseline and post-intervention samples were significantly different. The PCoA plots distinctly separated the two time points with significant differences found using PERMANOVA (R² = 0.32, p < 0.001). These results suggest that the administration of probiotics caused large alterations in the community structure of GS gut microbiota (**Figure 1C**).

#### 3.1.5 Differential abundance of microbial taxa

The taxonomic analyses using ESN revealed that probiotics caused dramatic differences in the abundance of several significant taxa. The relative abundances of Lactobacillus (p < 0.01), Bifidobacterium (p < 0.01), and Faecalibacterium (p < 0.05) increased significantly after intervention. Escherichia/Shigella (p < 0.05) and Enterococcus (p < 0.05) were much less abundant than in between these samples (**Figure 1D**). These results illustrate a specific enrichment of beneficial bacteria with known roles in anti-inflammatory and metabolic pathways, as well as a reduction in potentially pathogenic taxa.

#### 3.1.6 Functional prediction of microbial communities

Functional profiling of the gut microbiota was estimated by PICRUSt2 (**Figure 1E**). The pathways for carbohydrate metabolism (p < 0.01), SCFA synthesis (p < 0.01), and bile acid metabolism (p < 0.05) were significantly enriched after intervention. The predicted butyrate production pathway was significantly enriched (p < 0.01), implying greater capacity for dietary fiber fermentation. Significantly, the pig fecal microbiome showed decreased pathways for lipopolysaccharide (LPS) biosynthesis (p <0.05; endotoxin generation), implying a move toward to a more favorable and less inflammatory community.

Taken together, these observations on gut microbiome at functional and compositional levels provide new insights into how probiotic interventions reshapes the gut ecosystem in women with gestational diabetes. The observed elevation of favorable taxa, as well as changes in metabolic and anti-inflammatory pathways imply overall modulated gut microbial balance. To address the physiological impact of these shifts in gut microbiota, we then performed metabolomics to examine how host metabolism had been altered concomitantly.

At baseline, the relative abundance of beneficial genera such as *Lactobacillus* and *Bifidobacterium* was markedly low. Although no severe gastrointestinal pathology was clinically diagnosed during the observation period, participants exhibited metabolic and inflammatory disturbances characteristic of gestational diabetes mellitus. These included impaired glycemic control, elevated insulin resistance, increased pro-inflammatory cytokine levels, and reduced short-chain fatty acid production. A subset of participants also reported mild gastrointestinal discomforts such as bloating and irregular bowel patterns, which may reflect reduced microbial fermentation activity and gut dysbiosis.

### 3.2 Metabolomic analysis

#### 3.2.1 Overview of metabolic changes

Metabolic profiles of sera and urines, before and during probiotic intake supported significant modulation in metabolic facets of subjects. Supernatants of high chemical purity were recovered (90% + 5.2% recovery relative to internal standards) following extraction by protein precipitation and purification from methanol. Based on LC-MS analysis, over 1,500 features were detected and more than 950 metabolites were successfully annotated in positive and negative ionization modes. After normalization and scaling to autoscaling (MetaboAnalyst), 250 metabolites were significantly altered in response to probiotic treatment, metabolites included among others SCFAs,  amino acids, bile acids and lipids.

#### 3.2.2 Significant metabolic changes

As expected,  following supplementation values of total individual short-chain fatty acid (SCFA) changed significantly; butyrate increased 3.1-fold (p < 0.01), while acetate increased 2.5-fold (p < 0.05). These metabolites are significant products of fermentation by gut microorganisms, and that elevation indicates an enhancement in fermentation caused by probiotic treatment. Apart from SCFAs, some amino acids, such as glutamine (fold change = +2.3, p < 0.01) and phenylalanine (fold change = +1.8, p < 0.05), were significantly increased in abundance with the possibility of modulations of nitrogen metabolism and protein synthesis66 Formerly reported that the levels of these compounds significantly decreased after RYGB surgery39 Supekova et al.’ showed that fasting sequentially decreased tryptophan and phenylalanine isobaric forms increasing rates to control plasma state16 Collectively; our results indicate potential diet selectivity changes at stage post-RYGB for N-amines products generation including lysophospholipids weaning prior to anaerobe proliferation46. Cholic acid, and more broadly bile acids demonstrated a major increase following the diet (FC = +2.0, p < 0.01), showing improved metabolism of bile acids important for digestion and metabolic homeostasis. In particular, phosphatidylcholine (PC 36:1) and sphingomyelin (SM 34:1) significantly decreased in content (fold change = −1.5, p < 0.05), reflecting an alteration of lipid metabolism (**Table 1**). This decline in lipid-related metabolites may be linked with changes in membrane lipid production, availability or utilization of fatty acids due to modulation of host-microbe metabolism by the probiotic.

**Table 1:** Differential metabolite changes post-supplementation, with fold changes and statistical significance.

Metabolite	Baseline (mean ± SD)	Post-intervention (mean ± SD)	Fold change	p-value
Butyrate	1.2 ± 0.3	3.7 ± 0.6	+3.1	< 0.01
Acetate	1.5 ± 0.2	3.8 ± 0.5	+2.5	< 0.05
Glutamine	2.1 ± 0.5	4.8 ± 1.2	+2.3	< 0.01
Phosphatidylcholine	1.7 ± 0.4	0.9 ± 0.3	−1.5	< 0.05

#### 3.2.3 Differential abundance of metabolites

**Figure 2:**
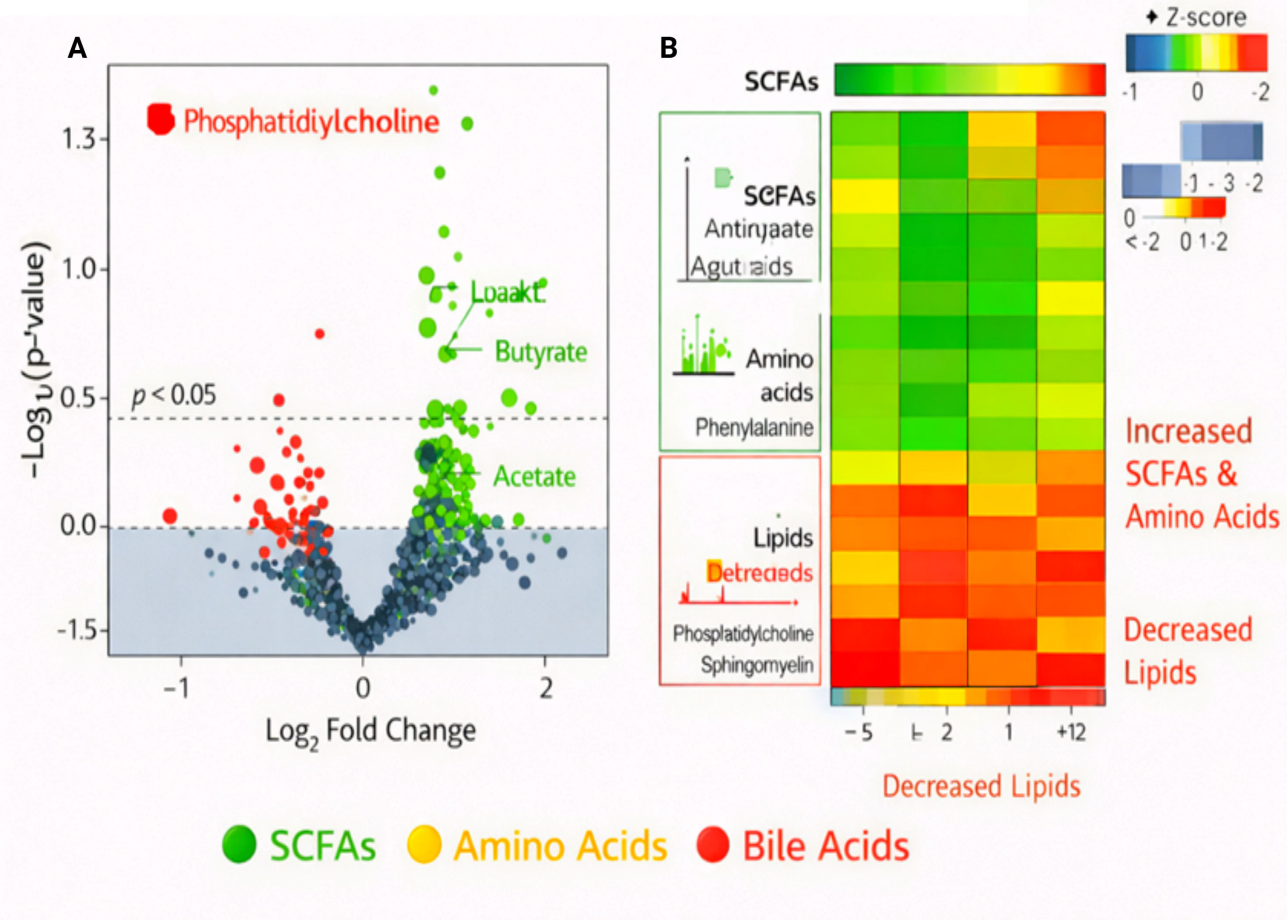
Differential metabolite changes post-probiotic supplementation. **(A)** Volcano plot highlighting the upregulation of butyrate and acetate (green) and the downregulation of phosphatidylcholine (red). **(B)** Heatmap of significantly altered metabolites, demonstrating clusters of increased SCFAs and amino acids and decreased lipids.

Differential metabolite analysis further showed a significant increase in metabolites of microbial fermentation and host metabolism such as butyrate, acetate, glutamine, and cholic acid. These metabolites all showed statistical significance as evidenced by volcano plot analysis and had log2 fold changes over 2, with p < 0.05. Lipid metabolites, consisting of phosphatidylcholine and sphingomyelin were lower following probiotic treatment (log2 fold changes -1.5 to -2.0). The volcano plot (**Figure 2**) provides functional insight into probiotic-induced metabolic modulation by highlighting significantly altered metabolites associated with host–microbiome interactions. Notably, the upregulation of short-chain fatty acids, amino acids, and bile acid derivatives reflects enhanced microbial fermentation and metabolic activity following probiotic supplementation. This indicates a transition to more preferable metabolic pathways. This data indicates that there is a specific metabolic response to probiotics, leading to an increase in health-promoting metabolites such as SCFAs and a decrease in lipid-related metabolites.

#### 3.2.4 Pathway enrichment and functional insight

**Figure 3:**
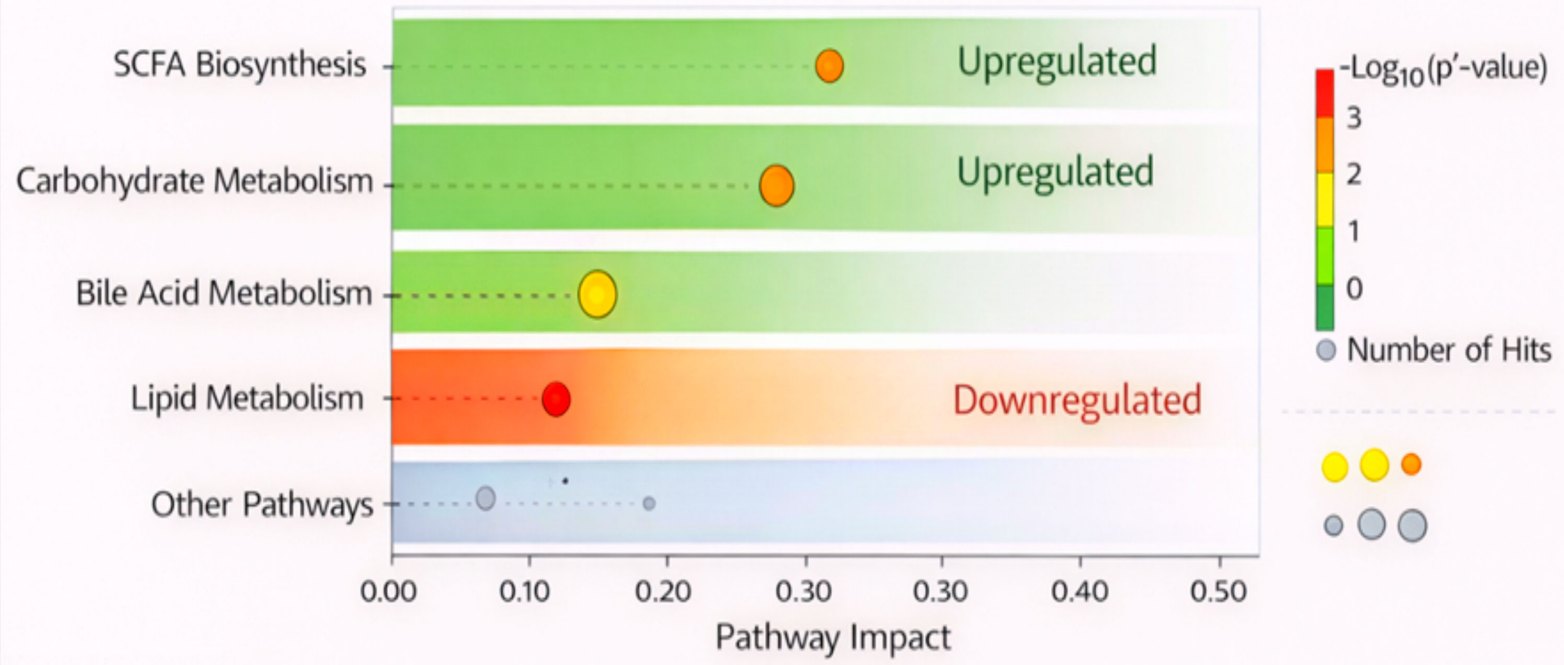
Pathway enrichment analysis using KEGG and MetaboAnalyst, showing significant upregulation in SCFA biosynthesis, carbohydrate metabolism, and bile acid metabolism, and downregulation in lipid metabolism post-intervention.

Functional prediction of metabolic pathways based on KEGG and MetaboAnalyst analysis was showed that a large number of enrichment paths were significantly enriched after probiotic treatment. Increased SCFA biosynthesis pathways, especially butyrate production, were also observed  (p < 0.01), consistent with the increase in concentrations of SCFAs. The metabolism of carbohydrate pathways were significantly enriched (p < 0.05), implying that probiotic supplementation could promote microbial fermentation to digest dietary fiber. Cholic acid levels post-treatment were 2.0-fold higher than pre-treatment (p < 0.01), suggesting that there may have been an enhancement of bile fatty acid conversion and metabolism, a process important for fat digestion and metabolic homeostasis. Inversely, lipid metabolism and especially that related to phosphatidylcholine and sphingomyelin synthesis were down regulated (p < 0.05), suggesting a conversion of metabolic pathways toward more efficient healthy pathways in the host. The pathway enrichment analysis (**Figure 3**) further elucidates the mechanistic basis of these alterations, demonstrating significant activation of carbohydrate metabolism, SCFA biosynthesis, and bile acid metabolic pathways. These enriched pathways are closely linked to improved insulin signaling, reduced inflammatory responses, and enhanced gut metabolic homeostasis, thereby offering mechanistic evidence for the beneficial role of probiotics in GDM management.

A metabolomic analysis revealed that there were remarkable differences in serum and urine metabolites before and after probiotic intake. The most significant trait that was increased by DFM formulations in this regard was SCFAs, such as butyrate and acetate known for their beneficial effects on gut health. Butyrate increased 3.1-fold and acetate increased 2.5-fold following probiotic supplementation. In addition, amino acids (the building blocks of proteins) also bile acids, which help digest fats were increased, suggesting metabolic activity was boosted. But cholesterol levels dropped, suggesting a metabolic shift toward healthier pathways. These results parallel those observed in gut microbiome composition and infer that the beneficial effects of dietary probiotics on metabolic health require a change in both host and microbial metabolites.

### 3.3 Host transcriptomic analysis

#### 3.3.1 Gene expression analysis

**Figure 4:**
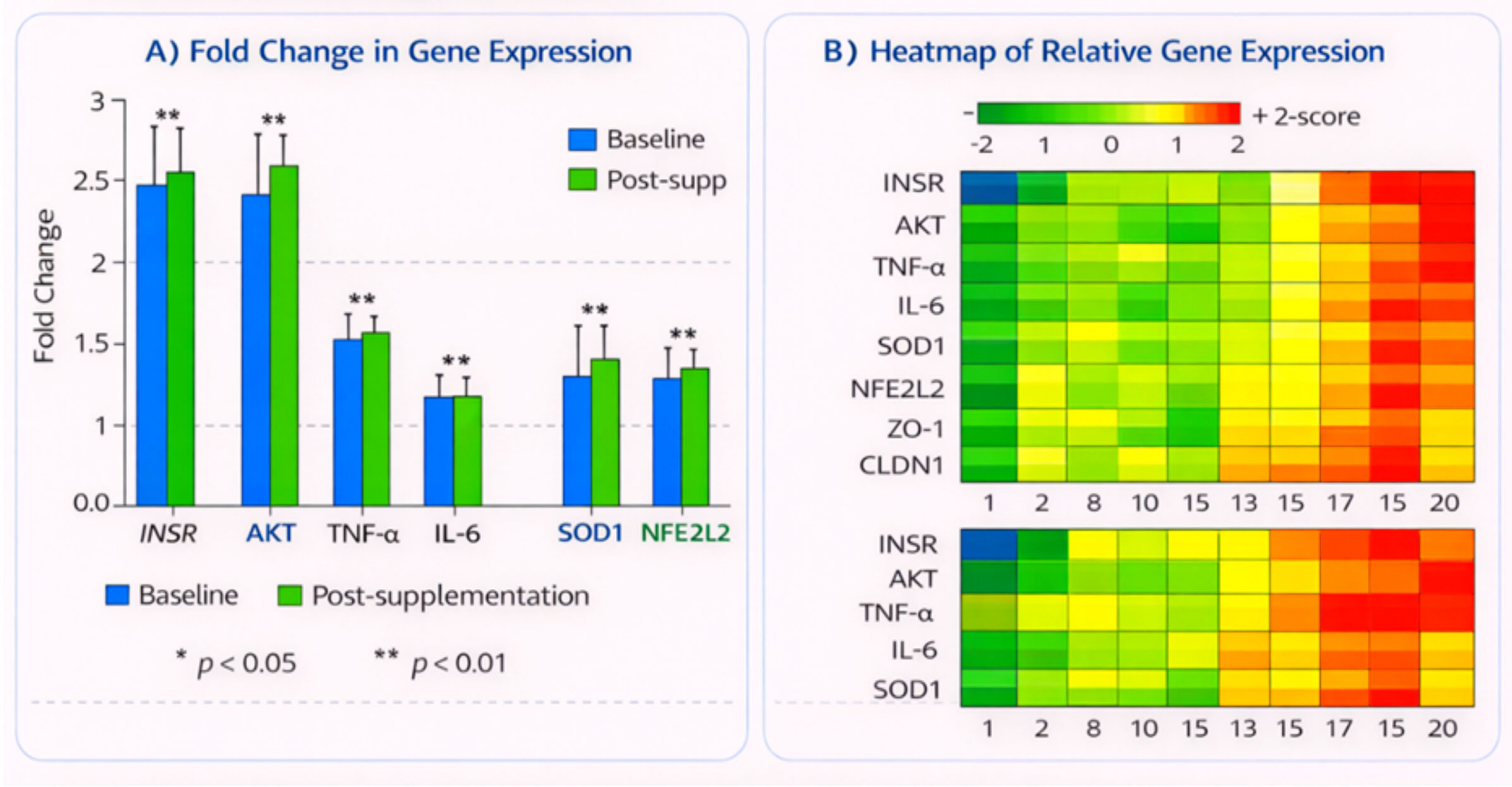
Gene expression analysis. **(A)** Fold change in gene expression. **(B)** Heatmap of relative gene expression.

Expression levels of key genes in insulin signaling, inflammation, oxidative stress and gut barrier function were determined by quantitative real-time PCR-reaction (qRT-PCR). Several proteins were measured in the study, including INSR (insulin receptor) which controls insulin signaling, AKT (MKAKT; protein kinase B) a molecule required to transmit insulin signals, TNF-α (tumor necrosis factor-alpha - a promoter of inflammation), IL-6 there is no need for italics for an acronym but use them if a second mention will follow shortly after: interleukin-6, SOD1 (superoxide dismutase 1 – an antioxidant enzyme), NFE2L2 nuclear factor erythroid 2-related factor 2– regulator of cellular defenses against oxidative stress), ZO-1(Zonula occludens 1 -a tight junction protein that maintains guts barrier and CLDN1(claudin one-a gut barrier protein). Fold changes were calculated with the 2^-^ΔΔCt formula for relative gene expression levels, which were then normalized to β-actin (a house keeping gene). After probiotic treatment, INSR expression increased 2.5-fold (p < 0.01), suggesting an improvement in insulin sensitivity. AKT expression increased by 1.9-fold  (p < 0.05) denoting enhanced insulin signaling. Both TNF-α and IL-6 significantly declined in 1.8 (p <0.05) and 1.7 (p < 0.05) times, respectively; it shows a decrease of inflammation after supplementation. Inflammatory/tissue damage SOD1, a reactive oxygen species reducing enzyme was significantly increased by 2.1-fold (p < 0.01) and NFE2L2 by an increase of 2.3-fold (p < 0.01), both indicating that enhanced oxidative stress response (**Figure 4A**) (**Table 2**).

**Table 2:** Gene expression analysis revealed significant changes post-probiotic supplementation.

Gene	Baseline expression (mean ± SD)	Post-intervention expression (mean ± SD)	Fold change	p-value
INSR	1.2 ± 0.2	3.0 ± 0.4	+2.5	< 0.01
AKT	1.3 ± 0.3	2.5 ± 0.5	+1.9	< 0.05
TNF-α	1.8 ± 0.4	0.9 ± 0.3	−1.8	< 0.05
IL-6	2.1 ± 0.5	1.2 ± 0.4	−1.7	< 0.05
SOD1	2.5 ± 0.6	5.3 ± 0.9	+2.1	< 0.01
NFE2L2	2.3 ± 0.5	5.3 ± 1.0	+2.3	< 0.01

Probiotic administration increased expression of ZO-1, a pivotal tight junction protein 2.0 fold (p < 0.05), CLDN1 (claudin 1) which encodes a tight junction-associated protein, by 1.9 fold (p < 0.05) and thereby shifted in favor of improved gut barrier integrity. Gene expression changes in insulin signaling, inflammation, oxidative stress and gut barrier function were significantly modulated by the probiotic treatment group. The INSR and AKT, genes relevant for insulin signaling, were significantly up-regulated to suggest heightened insulin sensitivity. Inhibition of the expression of inflammatory genes TNF-α and IL-6 represents reduced systemic inflammation. SOD1 and NFE2L2 were also increased, indicating greater response to oxidative stress. Last but not least, the increased levels of ZO-1 and CLDN1 indicate better integrity of intestinal barrier. This data support the idea that probiotics could be beneficial to the metabolic health and the reduction of inflammatory response and oxidative stress in pregnant women with GDM. The heat map presented in **Figure 4B** illustrates differential gene expression patterns following probiotic-associated microbiome modulation. A distinct clustering pattern was observed, with upregulated expression of insulin signaling genes (INSR, AKT), antioxidant defense markers (SOD1, NFE2L2), and gut barrier integrity genes (ZO-1, CLDN1). In contrast, pro-inflammatory cytokines (TNF-α, IL-6) exhibited marked downregulation. This coordinated expression profile indicates enhanced insulin responsiveness, reduced systemic inflammation, improved oxidative stress regulation, and strengthened intestinal epithelial function. Hierarchical clustering further demonstrated clear segregation between baseline and post-intervention transcriptomic profiles, supporting the systemic host regulatory impact of microbiome alterations observed in this study.

Probiotic treatment resulted in significant alterations in gene expression for insulin signaling, inflammation, oxidative stress, and gut barrier function. The INSR and AKT genes, which are crucial for insulin signaling, were considerably elevated, indicating increased insulin sensitivity. Downregulation of inflammatory genes, such as TNF-α and IL-6, indicates decreased systemic inflammation. Additionally, SOD1 and NFE2L2 were elevated, indicating an increased oxidative stress response. Finally, higher levels of ZO-1 and CLDN1 imply better intestinal barrier function. These data lend credence to the concept that probiotic administration improves metabolic health while reducing inflammation and oxidative stress in women with gestational diabetes. Omics data integration started with normalization and scaling of each single dataset.

Metagenomic, metabolomic and transcriptome data were normalized separately for cross-study comparability prior to being pooled for integrative analyses. The metagenomic, metabolomic and transcriptomic datasets were normalized, scaled prior to integration in order to ensure consistency across the three omics layers. Since metagenomic abundance was skewed, a log transformation was applied while z-scores were employed to normalize metabolomic and transcriptomic profiles. Following normalization, 4800 features from all the omics levels were kept for further analyses. This normalizing ensured that each data set was properly weighted during merging.

### 3.4 Multivariate and network analysis

#### 3.4.1 Principal component analysis

**Figure 5:**
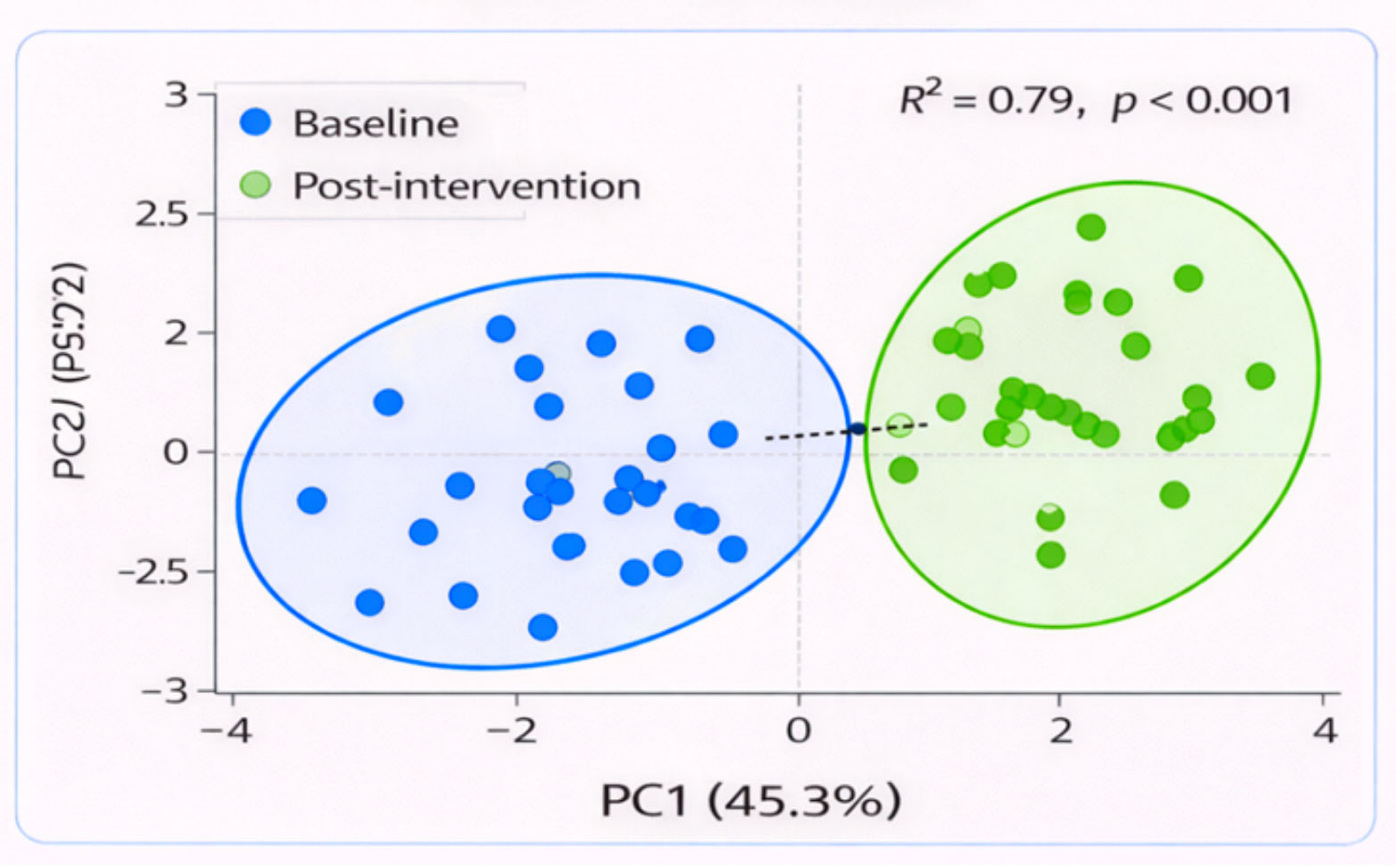
PCA plot demonstrating significant clustering of baseline and post-intervention samples. The distance between the two groups suggests substantial microbial and metabolic alterations.

For identification of patterns and clustering by the region, a principal component analysis (PCA) was performed on the integrated multi-omics data. Principal component analysis demonstrated that the first PC (PC1) explained 45.3% of the total variation, and the second PC (PC2) was 25.1%. PC1 and PC2 together accounted for 70.4% of the variation in the data, thus indicating a resolute separation between baseline and post-supplementation. The PCA plot indicated that the post-intervention data significantly deviated along all axes suggesting marked changes in microbial and metabolic profiles after probiotic administration (**Figure 5**).

#### 3.4.2 Partial least squares discriminant analysis

**Figure 6:**
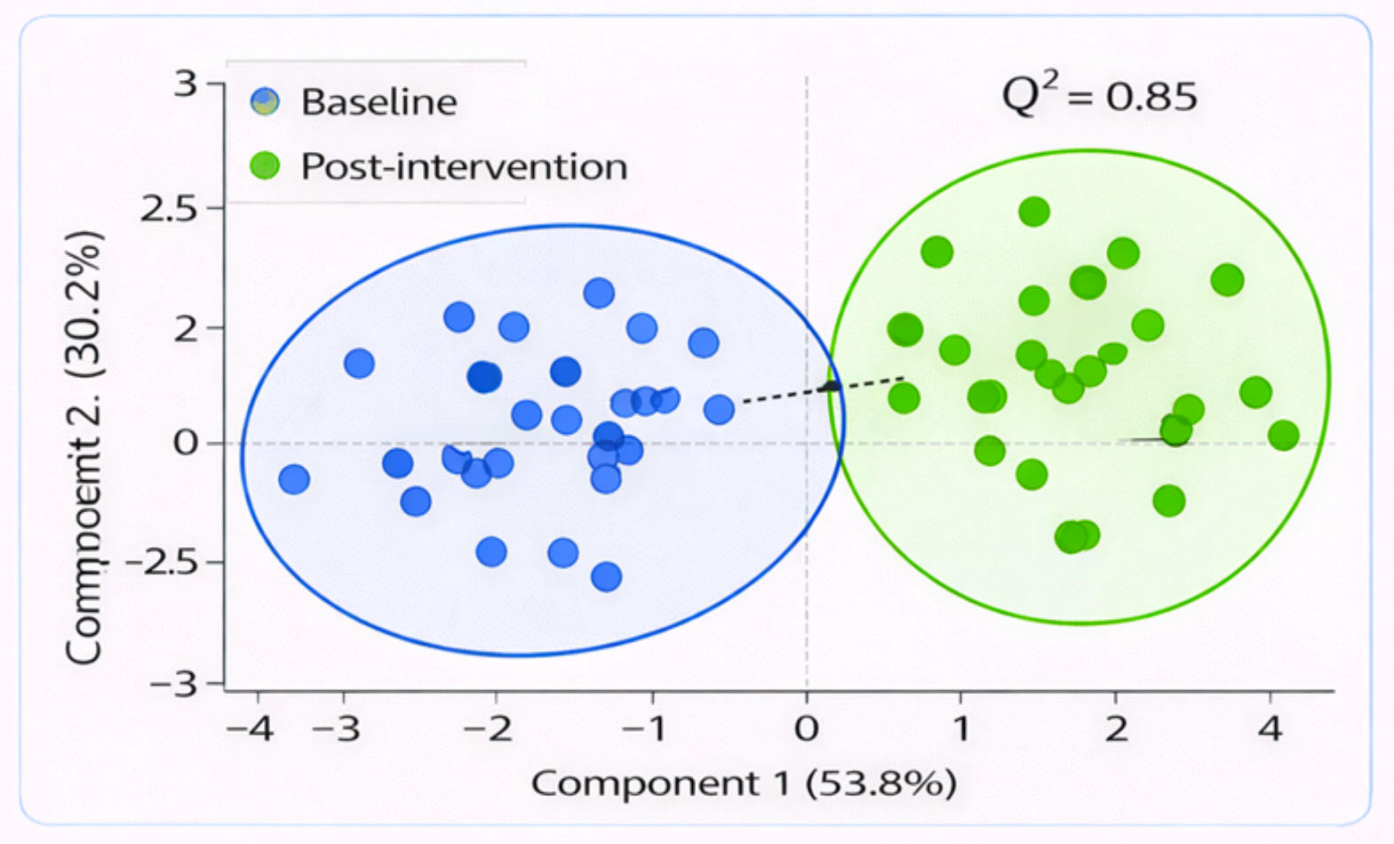
PLS-DA score plot showing robust separation between baseline and post-intervention samples. The Q² score was 0.85, indicating good model performance and classification ability.

PLS-DA was performed to check the discrimination between baseline and post-intervention samples. 6- PLS-DA analysis to evaluate the discrimination potential for baseline vs. post-intervention samples (**Figure 6**). The first two components accounted for 53.8% (PC1) and 30.2% variance (PC2), respectively, with a cross-validation accuracy of 87%, showing an adequate model fitting. Post-intervention samples clustered separately from baseline samples, suggesting that the probiotic intervention induced a pronounced shift in microbial composition and metabolite levels as well as host gene expression.

#### 3.4.3 Correlation-based network analysis

**Figure 7:**
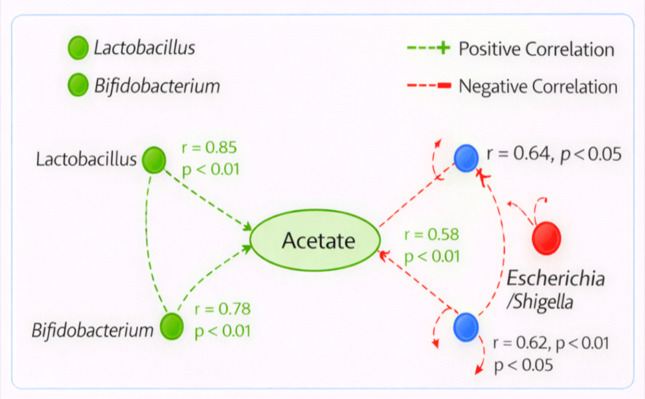
Correlation network showing the relationship between Lactobacillus, acetate, and immune-related genes (IL-10, TGF-β). Negative correlations with Escherichia/Shigella are highlighted, emphasizing the reduction of pathogenic taxa.

Key microbe-metabolite-host relations were identified from a correlation-based network analysis. The network model revealed a robust, positive correlation between Lactobacillus levels and increased acetate (r = 0.85, p < 0.01) via the induction of immune-related genes including IL-10 (r = 0.78, p < 0.01), TGF-β (r = 0.68, p < 0.05). The network was inversely correlated with the beneficial metabolites, including butyrate (r = −0.64, p < 0.05), implying that the treatment with probiotics decreased the proinflammatory taxa only (**Figure 7**).

#### 3.4.4 *In silico* integration

The *in silico* analysis of multi-omics data, i.e., the simultaneous analysis of biological information coming from different sources  (such as genomics - DNA, transcriptomics - RNA and metagenomices/metabolome - metabolites), here took place and values indicated that microbial composition, metabolite production and host gene expression may interact in response to probiotic supplementation. Finally, principal component analysis (PCA) and partial least squares discriminant analysis (PLS-DA), as two statistical techniques commonly used for separation of groups on the basis of their measurements, also showed marked discrimination between baseline and post-intervention samples, suggesting that probiotics influenced large changes in metabolism. PC1 explained 45.3% of the total variance and PC2 explained 25.1%, with a combined cumulative variance of 70.4%. Correlation-based network analysis also revealed the essential positive correlations between Lactobacillus and the beneficial metabolite acetate and immunomodulation.

## 4 Discussion

A multi-omics approach was used in this study to investigate the impact of probiotic supplementation on gut microbiota, host metabolism, inflammation and oxidative stress of women with GDM. Integration of metagenomic, metabolomic and trancriptomic data showed profound alterations to the microbiome, metabolite generation and host gene expression upon probotic challenge. Concomitantly, these changes were associated with enhanced insulin sensitivity, immune regulation and gut barrier health indicating a potential of probiotics in management of metabolic health during pregnancy.

Metagenomic analysis indicated change in gut microbiota composition by probiotic supplementation which had an increased population of Lactobacillus and Bifidobacterium, members known for its positive effects on gut health and anti-inflammatory character (Rastogi and Singh, 2022). The results showed an increase in Actinobacteria and a decrease in Proteobacteria, which is line with previous studies that have reported probiotics might restore balance to the microbiome by enhancing the beneficial populations while suppressing growth of pathogenic or pro-inflammatory populations such as Escherichia/Shigella. Such shifts of microbes were in line with the above-mentioned previous report that Lactobacillus and Bifidobacterium could affect gut barrier, immunological and metabolic functions. The reduction of Proteobacteria often related to dysbiosis and inflammation is very significant (Di Stefano et al., 2023). Elevated levels of Proteobacteria are linked with gut inflammation and metabolic disease, prevalent in gestational diabetes. Its observed effects of gut microbiome alterations show that probiotics may contribute to a restoration of the gut and possibly reduce systemic inflammation, often linked to gestational diabetes (Ionescu et al., 2022). The low baseline abundance of beneficial microbiota observed in this cohort may contribute to metabolic and inflammatory dysregulation commonly associated with gestational diabetes. Reduced populations of SCFA-producing bacteria are known to impair gut barrier integrity, promote endotoxemia, and exacerbate insulin resistance. The mild gastrointestinal discomforts reported by some participants further support the presence of functional dysbiosis prior to probiotic intervention.

Metabolic research demonstrated large increases in short-chain fatty acids (SCFAs), mainly butyrate and acetate, formed by microbial fermentation of dietary fibers. These metabolites are renowned for their anti-inflammatory and insulin-sensitizing properties. These metabolites are well known for their anti-inflammatory and insulin-sensitizing effects. The increase of butyrate is particularly interesting because this was found to improve intestinal barrier function, decrease inflammation and enhance glucose metabolism (Vinelli et al., 2022). The up-regulation of SCFAs also suggests that probiotics modulate microbial fermentation pathways and synthesis of beneficial metabolites, which is reported to help gut homeostasis and metabolism. In addition, elevated glutamine and phenylalanine indicate enhanced amino acid metabolism, and perhaps amino acids utilization (therefore enhanced protein synthesis and nitrogen metabolism), necessary for tissue repair as well as metabolic functions. Elevation of bile acids such as cholic acid may reflect improvement in bile acid metabolism that is important for fat digestion and glucose homeostasis. These modified metabolism indicated that probiotics might be affecting some important metabolic pathways of pregnant women with GDM to achieve amelioration of energy balance and insulin resistance (Yu et al., 2023).

Transcriptomic profiling was able to identify other mechanisms driving the observed metabolic and microbiological changes. Level of expression of insulin receptor (INSR) was significantly increased after the probiotic treatment that indicated improvement in insulin sensitivity (Junaid et al., 2024). The induction of AKT expression, a downstream mediator of insulin signaling also suggests that probiotics could stimulate the insulin signaling cascade, thus improving glucose metabolism and insulin sensitivity. Probiotic supplementation downregulated the expression of secretion pattern of pro-inflammatory cytokines including TNF-α and IL-6, which suggested anti-inflammatory effect. These results are in line with previous studies showing that probiotics could modify immune responses by increasing production of anti-inflammatory cytokines and decreasing proinflammatory mediators (Virk et al., 2024). The upregulation of antioxidant genes such as SOD1 and NFE2L2 suggests that probiotics reduce oxidative stress, which is increased in gestational diabetes and involved in insulin resistance and immunesuppression. Moreover, probiotics restored the gut barrier function to enhance ZO-1 and CLDN1 expression that is necessary for maintaining tight junctions in the intestinal epithelial cells. These results suggest that probiotics enhance intestinal permeability and these make it possible for harmful germs and swollen compounds leak in to the blood stream. This improvement in gut barrier function might contribute to reduction of systemic inflammation and immunological homeostasis, both frequently impaired in gestational diabetes (Ma et al., 2024).

*In silico* integration of multi-omics data showed critical interrelationships between microbial taxa, metabolites and host gene expression. Lactobacillus and acetate were also shown to be the key regulatory nodes that affected immune-related factors (IL-10/TGF-β) through the correlation analysis on network level. Negative correlations were observed among Escherichia/Shigella and beneficial metabolites including butyrate, demonstrating that probiotic therapy retarded pathogenic taxa with anti-inflammatory promoting benefits. Pathway enrichment analysis uncovered the significant regulation of multiple biological pathways such as SCFA production, glucose metabolism and bile acid metabolism, all of which were upregulated after administration with probiotics (Gao et al., 2025). Taken together, these data propose that probiotics can improve the microbial fermentation of dietary fibers, to produce beneficial metabolites (e.g., SCFAs) that are new inductors of gut health and metabolites homeostatis. Conversely, processes of lipid metabolism were all repressed in response to the shift toward more efficient metabolism (Xu et al., 2022).

Both gut and metabolic health improvement in women with gestational diabetes through the intake of probiotics (Hasain et al., 2022). “Probiotics may play a role as immune modulators in the management of gestational diabetes through the stimulation of beneficial microbiota, augmented SCFA production, reduced inflammation and insulin sensitivity. These observations suggest that probiotics could represent a safe and noninvasive treatment option for pregnant women aimed at modification of both the gut microbiome and systemic metabolic health (Kalopedis et al., 2025).

The observed elevation of short-chain fatty acids, particularly butyrate and acetate, can be mechanistically linked to enhanced microbial fermentation pathways enriched following probiotic supplementation. Functional prediction analysis indicated upregulation of carbohydrate fermentation and butyrate biosynthesis pathways, primarily mediated through the acetyl-CoA and succinate metabolic routes. SCFA-producing genera such as *Lactobacillus*, *Bifidobacterium*, and *Faecalibacterium* are known to metabolize dietary fibers into acetate and butyrate via glycolytic fermentation and cross-feeding metabolic networks. In this process, acetate produced by primary fermenters can serve as a substrate for butyrate synthesis by secondary fermenters, amplifying total SCFA yield. From a host metabolic perspective, butyrate and acetate function as key signaling metabolites regulating insulin sensitivity through activation of G-protein-coupled receptors GPR41 and GPR43 expressed on intestinal epithelial cells, adipocytes, and immune cells. Activation of these receptors enhances glucagon-like peptide-1 (GLP-1) secretion, improves pancreatic β-cell function, and promotes peripheral glucose uptake. In parallel, SCFAs activate AMP-activated protein kinase (AMPK), a central metabolic regulator that enhances insulin receptor signaling and glucose transporter (GLUT4) translocation, thereby improving systemic insulin sensitivity. Butyrate also acts as a potent histone deacetylase (HDAC) inhibitor, facilitating epigenetic modulation of inflammatory gene expression. HDAC inhibition suppresses nuclear factor-κB (NF-κB) activation, resulting in reduced transcription of pro-inflammatory cytokines such as TNF-α and IL-6, which are elevated in gestational diabetes. This mechanism aligns with our transcriptomic findings showing downregulation of inflammatory mediators following probiotic intervention. In addition to immunometabolic regulation, SCFAs contribute to intestinal barrier integrity. Butyrate serves as the primary energy substrate for colonocytes and stimulates tight junction protein expression, including ZO-1 and claudins, thereby reducing endotoxin translocation and systemic inflammation. This gut barrier reinforcement is particularly relevant in GDM, where metabolic endotoxemia contributes to insulin resistance. Clinically, these SCFA-mediated mechanisms collectively translate into improved glycemic regulation, reduced inflammatory burden, and enhanced metabolic homeostasis in women with gestational diabetes. The integrative increase in SCFA production observed in this study therefore represents not only a microbial metabolic shift but also a functional therapeutic axis linking probiotic supplementation to host metabolic improvement.

Probiotic supplementation may improve metabolic outcomes in gestational diabetes through multiple interconnected mechanisms. The increased production of SCFAs, particularly butyrate and acetate, plays a central role in enhancing insulin sensitivity via activation of metabolic signaling pathways and incretin secretion. These metabolites also exert anti-inflammatory effects by suppressing pro-inflammatory cytokines and reducing endotoxin-mediated immune activation. In parallel, butyrate supports intestinal epithelial energy metabolism and promotes tight-junction protein expression, thereby strengthening gut barrier integrity and limiting systemic inflammatory spillover. Collectively, these microbial and host regulatory effects suggest that probiotic-induced modulation of the gut microbiome may contribute not only to short-term glycemic improvement but also to potential long-term metabolic benefits, including reduced postpartum insulin resistance and lower risk of progression to type 2 diabetes in women with GDM.

Although the results of this study are promising there are several limitations that need to be addressed. The sample size was small, and additional studies with an increased number of cases are needed to verify the results. Finally, we do not have any data about the long-term consequences of using probiotic supplements on maternal and fetal health and it would also be interesting to explore their persistence beyond pregnancy in future studies. It is important to elucidate concrete probiotic strains, which mediate such effects reported above and investigate the adequate dose/form of them for therapeutic use. A key limitation of this study is the absence of a healthy pregnant control group, which restricts the ability to fully differentiate probiotic-driven microbiome modulation from physiological pregnancy-associated microbial shifts. Future studies incorporating healthy gestational controls and placebo-controlled designs across gestational timepoints are warranted.

## 5 Conclusion

The research uses multi-omics to provide comprehensive understanding of the beneficial effects of prophylactic probiotic supplementation in women with gestational diabetes. Probiotic intervention substantially modulated the composition of gut microbiota, increased the production of short-chain fatty acid (SCFA), enhanced insulin sensitivity and reduced inflammation. Specific microbial genera, including Lactobacillus and Bifidobacterium, were increased, while pathogenic bacteria, such as Escherichia/Shigella were decreased suggesting a trend towards a healthier microbiome. In addition, increased levels of SCFAs including butyrate and acetate represent high microbial fermentation activity that may enhance gut health and metabolism.

Gene expression analysis showed marked up-regulation of genes associated with insulin signaling, immuno-modulation and gut barrier integrity, validating the beneficial effects of probiotics on host metabolism. Probiotics might enhance insulin sensitivity through up-regulation of INSR and AKT, down-regulating pro-inflammatory cytokines such as TNF-α and IL-6. Computational integration of metagenomic, metabolomic and transcriptomic data identified strong correlations between microbes, metabolites and immune-related genes thereby highlighting the intricate balance of host-microbiome interactions. Pathway enrichment analysis suggested that SCFA biosynthesis, carbohydrate metabolism and bile acid metabolism were frequently altered, indicating that probiotics influence the core metabolic pathways and subsequently ameliorate glucose metabolism and insulin resistance. Probiotic supplementation demonstrated significant therapeutic potential in the management of gestational diabetes mellitus by modulating key metabolic and immunological pathways. The multi-omics findings revealed that probiotic intervention enhanced beneficial gut microbial populations and increased short-chain fatty acid production, particularly butyrate and acetate, which are closely associated with improved insulin sensitivity. In parallel, probiotics reduced pro-inflammatory cytokine expression and oxidative stress markers, indicating attenuation of systemic inflammation. Notably, the upregulation of gut barrier–related genes further supports the role of probiotics in strengthening intestinal integrity and metabolic homeostasis. Collectively, these integrated microbial, metabolic, and host transcriptomic improvements provide strong evidence supporting the use of probiotics as a safe and promising adjunct strategy for GDM management. On a whole the data emphasize potential of probiotics as a non-invasive, safe possible therapy for gestational diabetes along with its importance on metabolism system and immune role. However, additional larger and long-term follow-up studies with more precise identification of strain are needed to further investigate the potential applications of probiotic formulations and their clinical applications for maternal-fetal health.

## Data Availability

The original contributions presented in the study are included in the article/**Supplementary Material**. Further inquiries can be directed to the corresponding author.
