# Supplementary material for: Integrative multi-omics analysis reveals probiotic-induced microbiota shifts in women with gestational diabetes
Source: Front Cell Infect Microbiol. 2026 Mar 16;16:1782744. doi: 10.3389/fcimb.2026.1782744 (PMC13033712; doi:10.3389/fcimb.2026.1782744)
Supplement: Supplementary file 1 [file Table1.docx]

**Supplementary Table S1: Primer Sequences**

| **Gene** | **Forward Primer (5’ → 3’)** | **Reverse Primer (5’ → 3’)** | **Product Size (bp)** |
| --- | --- | --- | --- |
| **INSR** | AGGATGCTGTGGATGTGGAA | TCCAGGTGATGAGGTGTTGA | 142 |
| **AKT** | CCAGACTTGTGGTGGAGAAG | GCTGGTGTCAGATGTGGTTT | 135 |
| **TNF-α** | CCTCTCTCTAATCAGCCCTC | GAGGACCTGGGAGTAGATGA | 150 |
| **IL-6** | ACTCACCTCTTCAGAACGAA | TGGTCCTTAGCCACTCCTTC | 138 |
| **SOD1** | GGTGTGGCCGATGAAGAGAG | CCACAAGCCAAACACCAAAG | 120 |
| **NFE2L2** | TCCTCAACTGGATGTGGAAC | GGAATGGGAATGTCTCTGTG | 145 |
| **IL-10** | GACTTCTCCACAGGCACTGC | TCAGCTTCCTCTTCTGGCTC | 130 |
| **TGF-β** | CAGCACGTGGAGCTGTACTT | GGTTCGTGGATGTCTTGGTT | 148 |
| **ZO-1** | TGGTTGGCAGAGTTGAAGAG | CCAGGATGTCATAGTGGCTG | 140 |
| **CLDN1** | CCATGGCAACCAGTTTATGA | GGACTTGCAGGATGTAGGAA | 132 |
